# Meibomian gland dysfunction (MGD), as diagnosed by non-contact infrared Meibography, in dogs with ocular surface disorders (OSD): a retrospective study

**DOI:** 10.1186/s12917-019-2203-3

**Published:** 2019-12-05

**Authors:** Marta Viñas, Federica Maggio, Nunzio D’Anna, Roberto Rabozzi, Claudio Peruccio

**Affiliations:** 1 Policlinico Veterinario Roma Sud, Pilade Mazza 24, 00173 Rome, Italy; 2 Tufts Veterinary Emergency Treatment and Specialties, 525 South Str., Walpole, MA 02081 USA; 3 Turin Veterinary Centre (CVT), Lungo Dora Colletta 147, 10153 Torino, Italy

**Keywords:** Canine, Tear film, Lipid layer, Meibography, Interferometry, Ocular surface disorder, Meibomian gland dysfunction

## Abstract

**Background:**

Meibomian gland dysfunction (MGD) is one of the possible conditions underlying ocular surface disorders (OSD). Prevalence of MGD in dogs affected by OSD has not yet been reported. We aimed to evaluate the prevalence of MGD among OSD canine patients, which had been assessed by non-contact infrared meibography and interferometry, and to identify MGD associated factors that might guide its diagnosis. Medical records of canine patients examined for OSD between 2016 and 2019 were reviewed. The frequency of MGD was evaluated within different categories (skull conformation, gender, eye and STT-1). The putative MGD risk factors and frequency of MGD within grades of interferometry were evaluated in a regression analysis model and reported as odd ratios (ORs).

**Results:**

One hundred fifty eyes from 81 dogs with OSD were included with median age 75 months (range 3–192) and female representation with 52%. MGD was present in 70% of the examined eyes. MGD risk was higher in males OR_adj_ = 3.015 (95% CI: 1.395–6.514) (*P* = 0.005) and older patients OR_adj_ = 1.207 (95% CI: 1.081–1.348) (*P* = 0.001). No significant differences were found between left and right eyes (*P* = 0.66) or between the two types of skull conformation (*P* = 0.477) and MGD presence. MGD was associated to the lowest lipid layer (LL) thickness, as assessed by interferometry (grade 0) OR = 16.00 (95% CI: 2.104–121.68) (*P* < 0.001). STT values were not significantly associated with the presence of MGD (*P* > 0.05).

**Conclusions:**

MGD is a common underlying pathology in OSD. Being male and higher age are risk factors for MGD. An interferometry grade 0 may guide OSD diagnosis towards MGD.

## Background

Ocular surface homeostasis is maintained by the lacrimal functional unit, an integrated system comprising the lacrimal glands, ocular surface (cornea, conjunctiva and meibomian glands) and lids, and the sensory and motor nerves that connect them [1].

Any disorder in these structures can be classified as an ocular surface disorder (OSD), which includes also conditions like dry eye disease, and meibomian gland dysfunction (MGD) [2].

The main role of the lacrimal functional unit is to provide the ocular surface with an adequate and protective tear film (TF). The precorneal TF is composed of a thin superficial lipid layer (LL) and an underlying mucoaqueous layer, which occupies the bulk of the TF thickness and interacts directly with the glycocalyx of the epithelium via the membrane-spanning mucins [3, 4]. Lipids produced by the meibomian glands are the main component of the LL that prevents the evaporation of the aqueous phase and stabilizes it by lowering surface tension; thus, meibomian lipids are essential for the maintenance of ocular surface health and integrity [5].

MGD is a chronic, diffuse abnormality involving most of the meibomian glands [6], and characterized by terminal duct obstruction, retention of thickened opaque meibum with qualitative/quantitative changes, and cystic dilatation, shortening, atrophy or dropout (loss of acinar tissue) of the meibomiam glands [5]. Its prevalence in dogs has not yet been reported. MGD can be diagnosed by the assessment of meibomian glands with meibography; being the most recent non-contact technique, faster and easier to use than the contact ones, and suitable to be used on dogs [7].

The tear film-lipid layer (TF-LL) can be assessed by interferometry with the observation of interference patterns [8], which provide information on LL thickness and fluidity. Thick LLs show clear meshwork patterns with waves and interference fringes, while thinner layers are more homogeneous [9]. In veterinary medicine, the precorneal TF has been examined by polarized light biomicroscopy [10–12], and the surface lipid morphology in dogs has been 16 subdivided into different interference colors and 3 principal pattern variants (wave-like, islet and granitiform) [10].

The study aimed to retrospectively review the common meibographic and interferometric findings in the eyes of dogs affected by OSD to find out the proportion of cases with concomitant MGD, and to identify possible risk factors for MGD. In addition, the possible relationship of interferometry and MGD was studied.

## Results

### Patients’ characteristics

In a period of 3 years (2016–2019), 150 eyes from 81 dogs were examined. Median age was 75 months (range 3–192) and gender distribution was female for 52% (30% intact, 70% neutered) of eyes and male for 48% (100% entire). Twenty-seven different breeds were represented in this study with mixed-breed and English bulldog as the most frequent ones, with an equal representation of 12 dogs each. Brachycephalic breeds were represented by 59 eyes and non-brachycephalic by 91 eyes (Table 1).

**Table 1:** Demographic characteristics

	*N* = 150 eyes
Age (mo.)	75 [3–192]
Gender: Female	78 (52.0)
Skull: Non-Brachicephalic	91 (60.7)
Breeds	
Mixed-breeds	23 (15.3)
English Bulldog	22 (14.7)
Cavalier King Charles Spaniel	17 (11.3)
Poodle	18 (12.0)
Chihuahua	10 (6.7)
Shih Tzu	7 (4.7)
Jack Russell Terrier	6 (4.0)
Yorkshire Terrier	8 (5.3)
Labrador Retriever	2 (1.3)
Miniature Pinscher	4 (2.7)
Border Collie	4 (2.7)
Dachshund	3 (2.0)
Other breeds^a^	26 (17.3)

Continuous variables are expressed as median [range] and categorical variables as n eyes (%)

^a^ One single dog of the following breeds contributing with both eyes: Maltese, Pug, American Pit Bull Terrier, Australian Cattle dog, Basset Hound, Bolognese, Brittany Spaniel, Clumber Spaniel, English Springer Spaniel, Guadalupe Mastiff and Old English Sheepdog; and contributing with 1 eye: Australian Shepherd, Cane Corso, Coton de Tulear and Greyhound

### MGD prevalence and associated patients’ characteristics

MGD was diagnosed in 105 (70%) eyes, and it was the most common diagnosis among OSD eyes, followed by macroblepharon (16.7%) and exposure keratitis (12.7%) (Table 2).

**Table 2:** Clinical diagnosis of OSD

	Eyes n	(%)
MGD	105	(70)
Macroblepharon	25	(16.7)
Exposure Keratitis	19	(12.7)
Chronic keratitis	18	(12)
Keratoconjunctivitis sicca	16	(10.7)
Chronic conjunctivitis	12	(8)
Blepharitis	10	(6.7)
Blepharoconjunctivitis	10	(6.7)
Medial entropion	10	(6.7)
Trichiasis	10	(6.7)
Distichiasis	9	(6)
Caruncular trichiasis	7	(4.7)
Corneal pigmentation	4	(2.7)
Chalazion	3	(2)
Ectopic cilia	3	(2)
Eyelid mass	1	(0.7)

The sum of n is > 150, since eyes could suffer from more than one clinical diagnosis

*MGD* Meibomian Gland Dysfunction

No significant association was found between right or left eyes with regards to MGD presence (right eyes 34.7% vs. left eye 35.3%; *P* = 0.66).

Gender was a significant predictor for the presence of MGD, with three times more males than females presenting with MGD adjusted OR (OR_adj_) = 3.015 (95% CI: 1.395–6.514) (*P* = 0.005), while there was not difference between neutered or intact females presenting MGD OR = 1.122 (95% CI: 0.422–2.987) (*P* = 0.817). The risk of developing MGD increased with age with an estimated OR_adj_ of 1.207 (95% CI: 1.081–1.348) for any additional year (*P* = 0.001). However, no significant association was observed between skull conformation and MGD (*P* = 0.477) (Table 3).

**Table 3:** Association between independent predictors and MGD

Independent variable	OR _adj_	(95%CI)	*P*-value
Age	1.207	(1.081–1.348)	0.001
Gender			
Female	1.000	–	–
Male	3.015	(1.390–6.514)	0.005
Skull conformation			
Brachycephaly	1.000	–	–
Non-brachycephaly	1.346	(0.593–3.055)	0.477

*ORadj* OR adjusted for gender and age, *CI* confidence intervals, *MGD* Meibomian Glands Disorder, *1.000* Reference level

### Interferometry results and associated patients’ characteristics

Interferometry assessment showed that the most frequent grades were grade 1, present in 74 eyes (49.3%), followed by grade 2 (22%), grade 0 (19.4%), grade 3 (7.3%) and the last one grade 4 (2%).

**Fig. 1:**
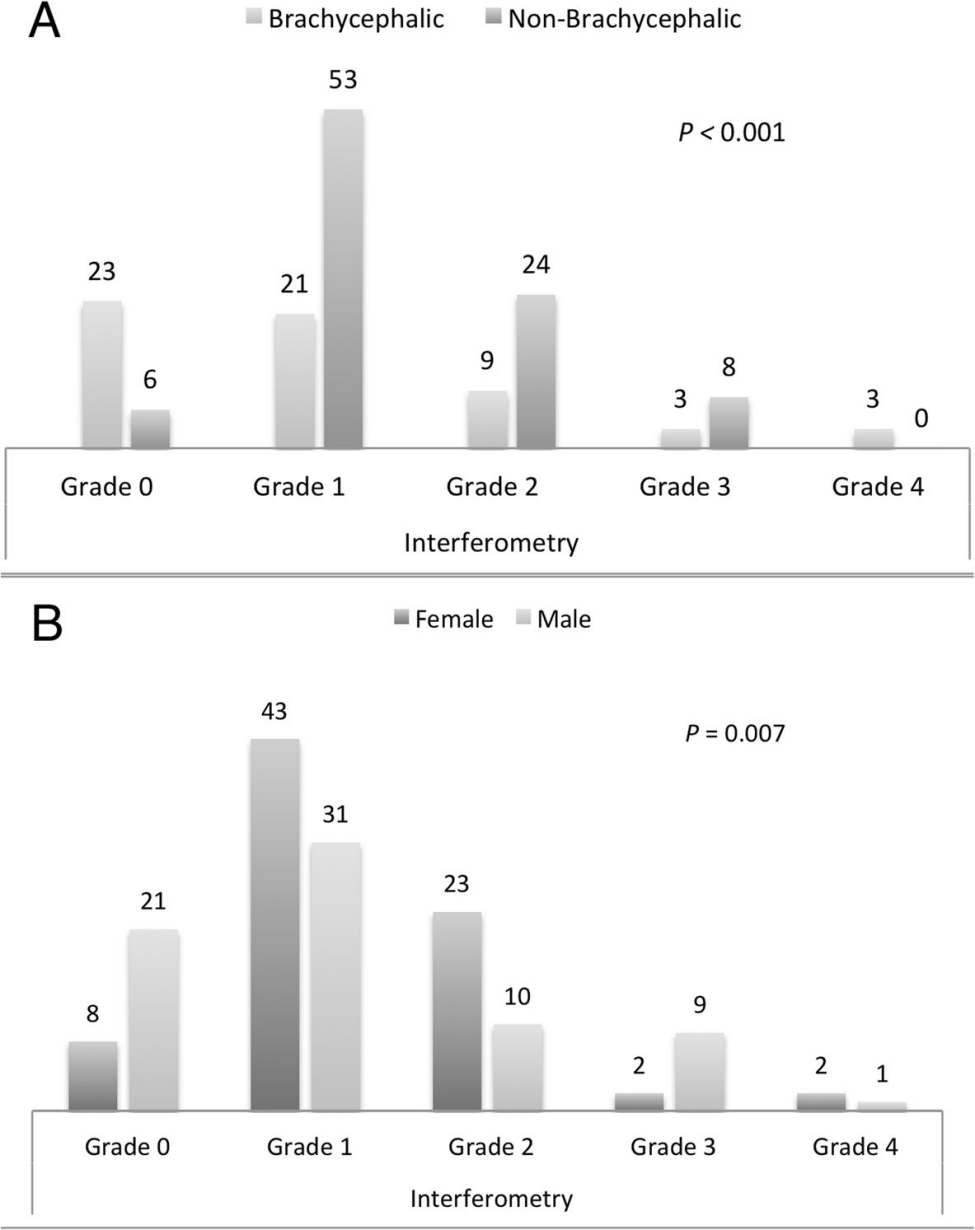
Distribution of skull conformation (**a**) and gender (**b**) within grades of interferometry

The distribution of skull conformation and gender within the grades of interferometry were significantly different (*P* < 0.001 and *P* = 0.007 respectively) (Fig. 1a, b**).**

The presence of MGD was significantly associated with interferometry grades (Table 4**)**. A higher risk of MGD was observed at grade 0 with thinner LL OR = 16.00 (95% CI: 2.104–121.68) (*P* < 0.001), and a lower risk was found for grade 2 OR = 0.414 (95% CI: 0.186–0.922) (*P* = 0.032) corresponding to thicker LL. The OR for grade 3 OR = 0.325 (95% CI: 0.094–1.127) (*P* = 0.078) was not significant, probably due to the small sample size (*n* = 11). Likewise, the OR of grade 4 was could not be assessed due to the small number of patients (*n* = 3), none of which had MGD. Thus, MGD incidence seems to decrease with increasing interferometry grades, as was suggested by the absence of the disease in grade 4**.**

**Table 4:** Association between presence of MGD and interferometry grades groups

Interferometry grades	OR	(95%CI)	*P*-value
Grade 0	16.00	(2.104–121.68)	< 0.001
Grade 1 (15–30 nm)	1.323	(0.656–2.669)	0.433
Grade 2 (31–60 nm)	0.414	(0.186–0.922)	0.032
Grade 3 (61–100 nm)	0.325	(0.094–1.127)	0.078
Grade 4 (> 100 nm)	NE	NE	NE

*OR* Odd ratios, *CI* Confidence intervals, *NE* Not evaluable

STT-1 values were not significantly associated with the presence of MGD and grades of interferometry (*P* > 0.05). In grade 1, eyes with MGD showed a STT-1 value of 22.7 mm/min (SD = 8.6) versus a value of 21.6 mm/min (SD = 8.6) in eyes free of MGD (*P* = 0.626). Also in grade 2, eyes with MGD showed a very similar STT-1 value when compared to eyes free of MGD [21.22 mm/min (SD = 4.7), 21.33 (SD = 5.1)] respectively (*P* = 0.948). In the remaining grades (grade 0, grade 3 and grade 4) the number of cases was too low to be analyzed.

## Discussion

Alteration of the TF is a frequent finding in any disorder affecting the lacrimal functional unit, and it is a common cause of ophthalmic examination in the dog.

The most common findings in our study were MGD (70%), followed by macroblepharon (16.7%) and eye exposure keratitis (12.7%), also considered potential causes of altered TF distribution.

Several techniques have been used to study the meibomian gland function. Slit-lamp biomicroscopy allows the assessment of the meibomian glands openings and may provide morphological evaluation of the glands in non-pigmented eyelids; however, it is limited as an indirect measure of meibomian gland structure and function [7]. Meibometry is a technique developed to measure basal meibum levels at the eyelid margin [7], and it has been used in dogs to quantify meibomian lipid secretion [13, 14]. However, a large range of meibometry values has been reported with low repeatability, and, thus, it is no longer considered clinically relevant in veterinary medicine [15]. Meibography is an in vivo technique that allows the visualization of meibomian gland morphology, including the glandular ducts and acini, and it may be performed by contact and non-contact techniques. The contact technique consists of the transillumination of the everted lid over a source of light allowing the visualization of the meibomian glands from the conjunctival surface of the eyelid [7]. In non-contact infrared meibography, the eyelids are everted and the meibomian glands observed by means of infrared light with no contact to the instrument [16]. In the medical records herein reviewed, non-contact meibography had been performed on all dogs, and thus, prevalence of MGD among OSD cases could be accurately estimated.

Meibography had been performed between the upper eyelid of the examined eyes. Human studies on MGD demonstrated no significant differences on examining either the upper or lower eyelid for MGD diagnosis, since when MGD was present, there was a good correlation between upper and lower meibomian gland loss [17]. Further studies are currently being conducted to find out if dogs with MGD are more frequently affected on the upper or lower eyelid.

The risk of developing MGD in humans increases with age, androgen deficiency and several other factors, including ophthalmic risk factors like dry eye and blepharitis [17–19]. However, according to a recent human study, gender has not been shown to be associated with MGD [19]. In the caseload of dogs presented herein, not only age but also gender was significantly associated to MGD, while not evidence was found regarding hormonal status (neutered vs intact).

Interferometry allows visualization of the kinetics of the oily layer of the TF that can be partially influenced by its composition, not only by its thickness [20–23]. LL composition, probably more than LL thickness, is highly correlated with the TF thinning rate caused by evaporation [24–26]. The current study shows that the presence of MGD is significantly associated with lower grade 0 of interferometry, while only in grade 4 no eyes were affected by MGD.

Brachycephalic dogs are reported to be prone to OSD [27]; however, in our study population no association between MGD and skull conformation was observed. Meibomian glands abnormalities in the brachicephalic breed Shih Tzu are more frequent in dogs with keratoconjunctivitis sicca than in control dogs [28]; thus, the association between skull conformation and OSD might include pathologies other than MGD.

In the presence of MGD, no association was observed between tear production (measured by STT-1) and grades of interferometry. A compensatory system has been described in humans by which the reduced oily layer of the TF, in case of MGD, is compensated by an increased secretion of aqueous component [23]. In the current study, we observed patients with MGD and a thin LL that can present a STT-1 > 15 mm/min, which could be explained by the above reported compensatory process. However, we also found eyes affected by MGD with thin TF-LL (grade 0 to 1) but low STT-1 (< 10 mm/min). Excessive thinning of the aqueous phase increases lipid contamination of the mucus layer present over the corneal surface epithelium, rendering it hydrophobic and less able to retain a stable TF [11], thus, in severe cases, the compensatory mechanism might not be sufficient. Otherwise, as described in humans [21], we observed patients not affected by MGD and with low STT-1 that can present a thick LL (61–100 nm), in this case a thicker LL could initially compensate the decreased function of the aqueous layer.

The balance of TF components is important for TF stability, and a compensatory system is thought to operate in response to changes in these components [23]. Further investigations, involving all components, are required to find out the mechanism of the TF compensatory system.

One limitation of our study is the inability to investigate goblet cells and their mucin secretion. Human studies speculate that an increase of TF stability by mucin production may require longer times [21].

In addition, it was not possible to standardize environmental important parameters, such as temperature and humidity [10]. Since all patients in our study have been examined at different times of the day, we can’t exclude little diurnal variation in tear secretion.

## Conclusion

MGD is a common underlying pathology in OSD. Being male and of older age are risk factors for MGD. A low grade of interferometry (grade 0) is associated with MGD, and thus, together with the presence of risk factors, might guide the OSD diagnosis towards MGD before confirmation by meibography.

## Methods

### Patients

Medical records of canine patients that had undergone TF examination after a diagnosis of OSD at the Ophthalmology Referrals Unit of the Turin Veterinary Centre, Italy from 2016 to 2019 were reviewed. Informed consent form was obtained from the owners for the use of the patient data. Diagnosis of OSD was made after a complete ophthalmic examination and was based on clinical signs of loss of ocular surface homeostasis caused by anatomical and/or functional alterations of one or more components of the lacrimal functional unit including: the ocular surface (cornea and conjunctiva) with sensory nerve endings, the afferent and efferent innervation to stimulate tear secretion, the lacrimal gland, the meibomian gland, the conjunctival goblet cells and the eyelids.

Information from each case included gender, breed, skull conformation (divided into brachycephalic and non-brachycephalic skull), age, eye examined (left or right), concurrent ocular diseases, meibography and interferometry.

### Ophthalmic examination

All dogs in this study had undergone a complete and bilateral ophthalmic examination by a board-certified veterinary ophthalmologist. The examination included assessments of the palpebral reflex, menace response, pupillary light and dazzle reflexes, Schirmer tear test 1 (STT-1) (Eickemeyer®; Tuttlingen, Germany), slit-lamp biomicroscopy (Keeler®; PSL Classic, Windsor, Berkshire, UK), fluorescein staining (Optitech® eyecare; An-Vision, Hennigsdorf, Germany), applanation tonometry (Tono-Pen Vet®; Reichert Technologies, Depew, NY, USA), and indirect ophthalmoscopy (Omega® 2000; Heine, Herrsching, Germany).

Patients that were on any kind of OSD treatment met inclusion criteria only if treatment had been suspended ≥2 days before performing the TF examination. Records of dogs with orbital or nictitating membrane disorders, with intraocular diseases (i.e. uveitis, cataract, glaucoma, retinal detachment), or with a recent history of surgery (less than 1 month) were excluded from the study.

### TF examination

**Fig. 2:**
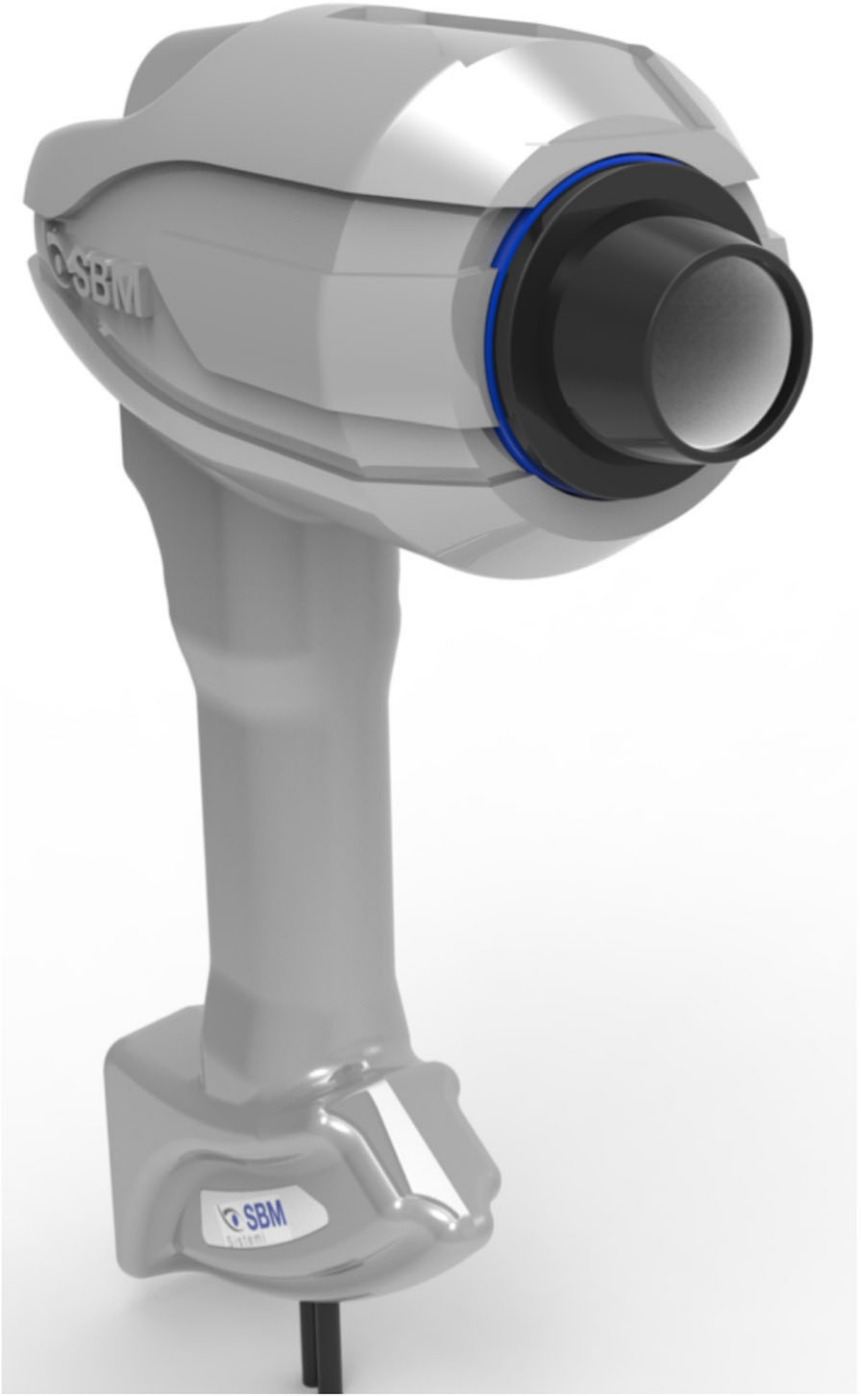
SBM Sistemi, portable instrument for meibography and interferometry. Copyright SBM Sistemi. Reproduced with permission

A hand-held ocular surface analyzer (OSA-VET®, SBM Sistemi, Torino, Italy), equipped with infrared and white led lights for meibography and interferometry, respectively, was used to perform non-contact infrared meibography of the upper lid and interferometry **(**Fig. 2**).**

**Fig. 3:**
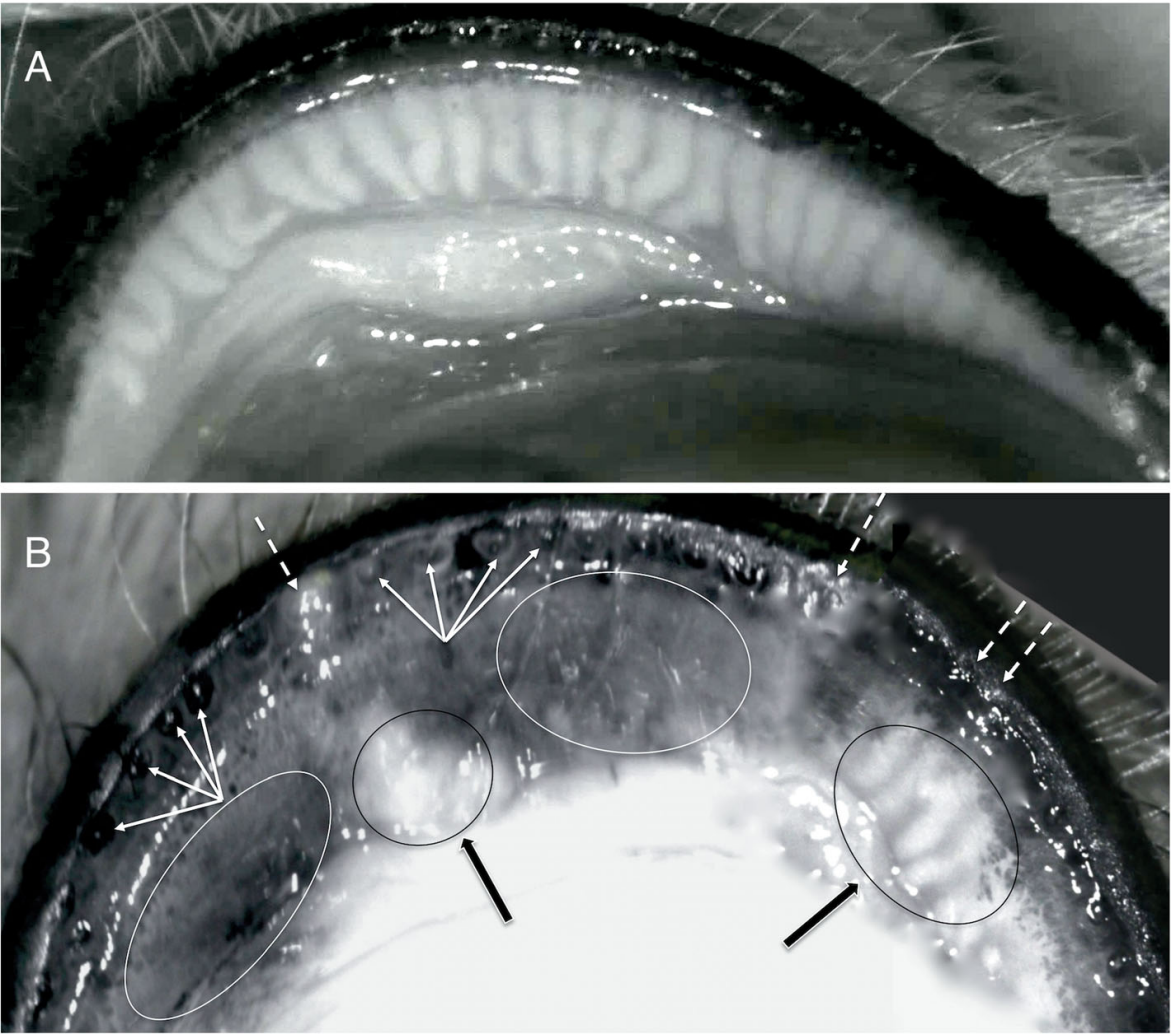
Non-contact meibography. **a** Non-contact infrared meibography of an upper eyelid with normal meibomian glands. **b** An upper eyelid affected by MGD. The eyelid margin is characterized by diffused ductal occlusion and plugging of the MGs orifices (dotted arrows). The eyelid mucocutaneous junction is moved posteriorly with retroplacement of ductal openings (multiple arrows). Areas characterized by cystic dilatation of the ducts filled with extremely dense opaque secretion (black arrows and ovals) alternate with darker areas due to the diffused atrophy of the acini detected as gland dropout on infrared meibography (white ovals)

Meibography was mainly directed at the diagnosis of MGD, and only 2 categories were considered: MGD affected or unaffected. A diagnosis of MGD was achieved when terminal duct obstruction was present along over 75% of the eyelid margins and/or when meibomian glands dilatation, shortening, atrophy or dropout affected more than 50% of the eyelid [5]. (Fig. 3**)**.

Meibomian gland expression has been used in some cases to evaluate the color and consistency of the expressed meibum or to confirm ductal occlusion.

**Fig. 4:**
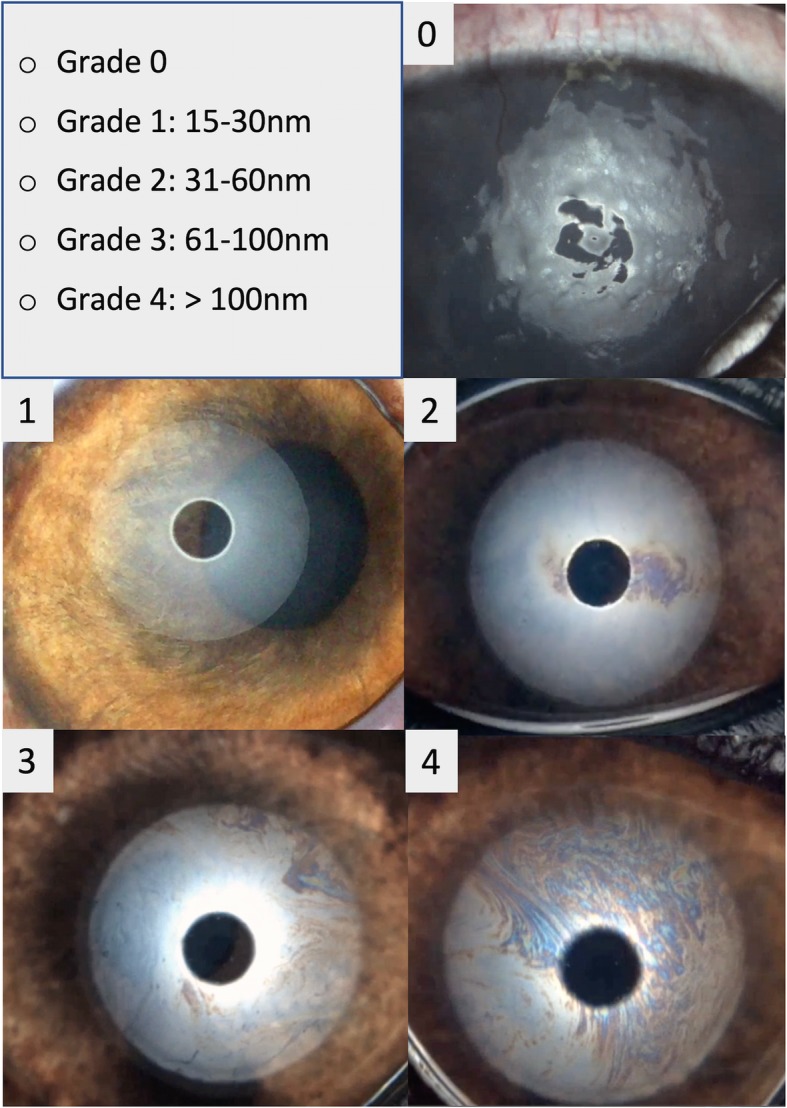
Grading scale of interferometric patterns. A four-interval grading scale of interferometric patterns was adopted from human literature and one more grade (grade 0) was added to evaluate lipid layer (LL) thickness in dogs. Grade 0 includes cases of almost complete absence of the aqueous phase, with lipid-contaminated mucus over the surface of the corneal epithelium. Grade 1 (15–30 nm) corresponds to faintly visible homogeneous meshwork pattern; grade 2 (31–60 nm), when a more compact meshwork pattern with grey waves and occasional colored shades is observed; grade 3 (61–100 nm), when a meshwork with waves and interference fringes with some colors are noted and grade 4 (more than 100 nm), when waves with many colors are present

Interferometry was used to assess the TF-LL patterns, each one reflecting a specific thickness of the TF-LL. The patterns were classified according to a grading scale recommended by the instrument manufacturer, with categories adopted for humans [8, 9, 29], and adapted for veterinary use, according to the available literature [10–12]. A five-interval scale was used as follows (Fig. 4): Grade 0 included cases of almost complete absence of the aqueous phase, with lipid-contaminated mucus over the surface of the corneal epithelium; this grade was added since, without a liquid component present on the cornea it was impossible to evaluate the lipids, sometimes scattered over the ocular surface in static colored islets. Grade 1 (15–30 nm), when faintly visible homogeneous meshwork pattern was present; grade 2 (31–60 nm), when a more compact meshwork pattern with grey waves was observed; grade 3 (61–100 nm), when a meshwork with waves and interference fringes with some colors was noted and grade 4 (> 100 nm), when waves with many colors were present.

STT-1 was used to assess tear production. The continuous variable was subdivided into three categories: < 10 mm/min, 10–15 mm/min and > 15 mm/min.

In order to avoid excessive inter-observer differences in the assignment of ranks the interferometry was done by the same experienced operator.

The data were retrospectively collected and reviewed, and elaborated for statistical analysis.

### Statistical analysis

Normality of the underlying distribution of the continuous variables was assessed using the Shapiro-Wilk test.

Continuous variables with normal distribution were presented as mean and standard deviation (SD), and those with non-normal distribution were presented as median (range), while absolute frequencies and percentage were used for categorical variables.

The MGD frequency distribution was evaluated within different categories (skull conformation, gender, eye and STT-1) using the Chi-squared test.

Independent variables (gender, age, skull conformation) were evaluated as risk factors for developing MGD (dependent variable) in a logistic regression model and reported as odd ratios (ORs) with their 95% confidence (95% CI) intervals. ORs were adjusted by gender and age. The skull conformation was considered as a categorical variable and brachycephaly was used as the reference level to which nonbrachycephaly was compared. Association between the presence of MGD and interferometry grades was analyzed by ORs.

Probability values of < 0.05 were considered significant.

Statistical analysis was performed with R software (version 3.4.3. R project, Auckland, New Zealand) and MedCalc software (version 19.0.6 Bvba, Ostend, Belgium).

## Data Availability

All data generated or analyzed during this study will be available from the corresponding author on reasonable request.

## Abbreviations

LL: Lipid layer
MGD: Meibomian gland dysfunction
OSD: Ocular surface disorders
STT-1: Schirmer tear test-1
TF: Tear film
TF-LL: Tear film-lipid layer
